# Glatiramer acetate stimulates phagocytosis and intracellular killing of *Escherichia coli* by macrophages and microglial cells

**DOI:** 10.3389/fimmu.2026.1761044

**Published:** 2026-03-09

**Authors:** Jana Seele, Darius Häusler, Roxana Heidemann, Martin S. Weber, Roland Nau

**Affiliations:** 1 Department of Geriatrics, Evangelisches Krankenhaus Göttingen-Weende, Göttingen, Germany; 2 Department of Neuropathology, University Medical Center Göttingen, Georg-August-University Göttingen, Göttingen, Germany; 3 Fraunhofer-Institute for Translational Medicine and Pharmacology (ITMP), Göttingen, Germany; 4 Department of Neurology, University Medical Center Göttingen, Göttingen, Germany

**Keywords:** bactericidal activity, *Escherichia coli*, glatiramer acetate, macrophages, microglia, phagocytosis

## Abstract

**Background:**

In contrast to other medications used for the treatment of multiple sclerosis (MS), glatiramer acetate (GA), a synthetic polypeptide, has not been associated with an increased risk of infections. We studied the effect of GA on innate immune cells and its ability to ingest and kill bacteria.

**Methods:**

GA was co-incubated with peritoneal macrophages and microglial cells from wild-type C57BL/6 and interleukin (IL)-10^-/-^ mice. Subsequently, phagocytosis and intracellular killing of encapsulated *Escherichia* (*E*.) *coli* K1 by these phagocytes was analyzed. Using microglia from wild-type mice, IL10 was blocked by the addition of antibodies. GA was administered *in vivo* to wild-type and IL10^-/-^ mice. Afterwards, peritoneal macrophages were isolated and tested for their phagocytic capacity.

**Results:**

GA increased phagocytosis in a concentration- and time-dependent manner of *E. coli* by macrophages and microglia isolated from wild-type mice. GA also increases the intracellular killing of *E. coli*. Macrophages from IL10-/- mice were one order of magnitude less susceptible to GA. Blocking of IL10 reduced phagocytosis in microglia in a concentration-dependent manner. GA stimulated phagocytosis also in macrophages from old mice. The *in vivo* administration of GA increased the phagocytic capacity of wild-type but not IL10-/- macrophages *ex vivo*.

**Conclusions:**

As a consequence of the increased threat by multi-resistant bacteria, immunomodulators with few side effects, which are able to stimulate the phagocytosis and killing of bacteria, are highly desirable. GA stimulates the phagocytosis and intracellular killing of pathogenic bacteria. Due to its low toxicity even during long-term treatment, GA is an excellent candidate to increase the resistance of patients to infection, potentially reducing the amount of antibiotics prescribed.

## Introduction

As a consequence of their disability and of the use of disease-modifying drugs, patients with multiple sclerosis (MS) have an increased risk of infections (bacterial, viral, fungal, or parasitic) in comparison to the general population (1). Many agents used for the treatment of acute MS attacks or for the reduction of the frequency of relapses and the progression of disability in MS impair immune surveillance and may increase the risk of patients to develop community-acquired or opportunistic infections including central nervous system (CNS) infections (2). Special attention has focused on progressive multifocal leukoencephalopathy (PML) caused by JC virus. PML is a rare complication of patients with hematologic malignancies with an incidence of <0.3 per 100,000 persons/year in the general population. The estimated incidence of PML in MS patients treated with natalizumab is 2.1 cases per 1,000 patients/year (3, 4). PML induction has also been observed after the administration of the monoclonal antibodies rituximab, efalizumab, infliximab, or alemtuzumab (5). Because of the aging of the immune system (“immunosenescence”), older patients possess an increased susceptibility to infections (6), which is aggravated by immunosuppressive drugs. A treatment option for older or/and immunosuppressed MS patients which does not affect the phagocytosis of bacteria or may even enhance it would be highly desirable.

Glatiramer acetate (GA), a mixed random polymer of alanine, glutamate, lysine, and tyrosine, was traditionally believed to act directly on T lymphocytes, inducing a shift toward T-helper-2 (Th2) lymphocytes. More recent work demonstrated an influence of GA on antigen-presenting cells, in particular monocytes and dendritic cells, i.e., GA has broad immunomodulatory properties influencing both the innate and adaptive immune system (7, 8). GA inhibited the production of IFN-β and promoted the differentiation of monocytes into an anti-inflammatory M2 phenotype by a MyD88-independent pathway (9, 10). GA diminished transitional B-cell density, reduced the expression of CD69, CD25, and CD95, decreased TNFα secretion, but increased IL-10 release (11).

The use of GA has not been associated with an increased risk of infections (12). In clinical trials, the frequency of infections in patients receiving daily Copaxone™ (*n* = 563) was 30% compared to 28% in patients receiving placebo (*n* = 564) (13). In a murine model of cerebral toxoplasmosis, the number of parasites in the brains of GA-treated animals was not increased. Conversely, the number of parasite cysts more than doubled in fingolimod-treated mice (14). In mice infected intraperitoneally with *Plasmodium berghei*, GA treatment resulted in a lower risk of developing cerebral malaria (57.7% versus 84.6%). GA did not influence parasitemia. The mechanism of action appeared to be a reduction of interferon (IFN)-γ release and a subsequent lowering of the leukocyte infiltration of the brain (15). In murine HIV-1 encephalitis (HIVE), GA treatment had anti-inflammatory and neuroprotective properties consisting in reduced micro- and astrogliosis, increased expression of interleukin-10 and brain-derived neurotrophic factor, and reduced expression of inducible nitric oxide synthase (16).

GA does not appear to be an immunosuppressant. The influence of GA on the phagocytosis and intracellular inactivation of living pathogens, however, has not been studied. The present study was performed to assess the effect of GA on the ability of phagocytes to ingest and kill bacteria.

## Materials and methods

Glatiramer acetate (GA): GA (Copaxone™, molecular weight 5–9 kDa) was obtained from Teva GmbH (Ulm, Germany). It was diluted in phosphate-buffered saline (PBS) to the concentrations used in the individual experiments.

IL10 antibodies: IL-10 was neutralized by adding 0.01–100 ng/mL monoclonal rat IgG1 anti-IL-10 antibody (clone: JES5-2A5; BioXcell, BIOZOL Diagnostica, Eching, Germany) or 100 ng/mL isotype control antibody (clone: TNP6A7; BioXcell) to the cell culture supernatants.

Bacteria: An *Escherichia coli* serotype O18:K1:H7 strain originally isolated from the CSF of a neonate with meningitis was kindly provided by Dr. G. Zysk, Institute for Medical Microbiology and Virology, Heinrich-Heine-Universität Düsseldorf, Germany (17–19).

Mice: Female young adult C57BL/6 mice (2–4 months, mean age 10.5 weeks) and old mice (20 months) were purchased from Charles River (Wilmington, USA) and kept at the Central Animal Care Facility, University Medicine Göttingen (UMG), at a day–night cycle of 12 h, constant room temperature of 20°C, and moisture of 55% with free access to food and water. The C57BL/6 mice were bred at the Central Animal Care Facility, UMG. Newborn mice (days 1–3) of both sexes were used for the preparation of microglial cells.

IL10-deficient mice: IL-10 knockout mice (#004368) on a C57BL/6 background were purchased from Jackson Laboratory, Bar Harbor, ME, USA.

Primary macrophage cultures: The mice were killed by CO_2_ using 100% CO_2_ at a flow rate of 50% of the chamber volume per minute. Thereafter, the peritoneal cavity was rinsed with 20 mL of PBS. The cell suspension was washed with PBS by centrifugation at 500 *x g* for 8 min. The cells were resuspended in Dulbecco’s Modified Eagle’s Medium (DMEM) supplemented with 10% fetal calf serum (FCS), 100 U/mL penicillin, and 100 µg/mL streptomycin. The macrophages were seeded at a density of 150,000 cells per well in a 96-well plate. These plates were incubated for 48 h at 37°C, before the phagocytosis assay was started.

Primary microglial cultures: Microglial cells were prepared from the brains of newborn C57BL/6 mice after they had been decapitated under CO_2_ anesthesia. After removal of the meninges, the cells were mechanically dissociated and suspended in Dulbecco’s modified Eagle’s medium (DMEM) (Gibco, Karlsruhe, Germany) supplemented with 10% fetal calf serum (FCS), 100 U/mL penicillin, and 100 μg/mL streptomycin. The cells were plated at a density of two brains per T75 culture flask (Corning Costar GmbH, Wiesbaden, Germany) and incubated at 37°C in a humid atmosphere with 5% CO_2_. After 10 days of cultivation, microglial growth was stimulated by the addition of 1/3 cell culture supernatant of the cell line L929. After an additional 3 to 4 days, the microglial cells were separated from the confluent astrocyte layer by shaking 200×/min for 30 min and plated in 96-well plates at a density of 50,000 cells/well for phagocytosis experiments.

Cultivation of L929 cells: Cultivation of L929 cells (ATTC^®^: CCL-1) and preparation of the cell culture supernatants were performed as described (20).

Quantification of phagocytosis and intracellular killing: For the construction of dose–response curves, primary cultures of peritoneal macrophages and microglial cells were incubated with GA diluted in PBS at concentrations of 1, 3, 10, 30, 100, and 300 µg/mL for 24 h at 37°C. To determine the optimum duration of GA stimulation, the macrophages were exposed to GA for 1, 5, 24, or 120 h. Stimulation with 0.01 µg/mL lipopolysaccharide (LPS; from *E. coli*, Sigma-Aldrich, Schnelldorf, Germany) dissolved in PBS for 24 h served as a positive control and for 24 h of an equal volume of PBS as a negative control. After stimulation, the macrophages or microglia were co-incubated with *E. coli* K1 for 60 min. Then, the samples were washed with PBS, and extracellular bacteria were killed by gentamicin at a concentration of 100 µg/mL for 60 min (experiments studying phagocytosis) or 60–240 min (experiments studying intracellular killing). At the respective time point, the phagocytes were washed with PBS and lysed by the addition of distilled water. The resulting suspension was quantitatively plated on blood agar plates. The *E. coli* colonies grown on the plates were counted after 16 h of incubation at 37°C. For the assessment of the rate of phagocytosis, the number of intracellular bacteria was determined after 60 min of gentamicin treatment. For the assessment of intracellular killing, the number of intracellular bacteria was quantified at 60, 120, and 180 min after co-incubation of phagocytes containing internalized *E. coli* K1 in the presence of extracellular gentamicin. To ensure the inter-day comparability of individual experiments, the median number of phagocytosed bacteria in the unstimulated control wells of each experiment after 60 min of phagocytosis followed by 60 min of gentamicin treatment to kill extracellularly situated bacteria was set as 100%, and the number of phagocytosed bacteria in each individual well was expressed as %.

*In vivo* treatment of C57BL/6 and IL10^-/-^ mice with GA: In order to assess whether macrophages can be primed by GA *in vivo* and whether this effect required IL10, the mice received subcutaneous (s.c.) injections of 150 µg GA/mouse once daily for five consecutive days. They were then killed by using CO_2_, and macrophages were prepared and further processed as described. The phagocytic activity of macrophages from mice primed by GA *in vivo* was compared with that of macrophages from mice which received equal volumes of s.c. saline. This animal experiment was approved by the Animal Care Committee of the University Medical Center Göttingen (UMG) and by the Niedersächsisches Landesamt für Verbraucherschutz und Lebensmittelsicherheit (LAVES), Braunschweig, Lower Saxony, Germany.

Measurement of cytokine concentrations: The concentrations of the cytokines TNFα, IL12/IL23 p40, IL12 p70, and γ-interferon in the supernatants of cultured macrophages were determined by sandwich enzyme immunoassay according to the manufacturer’s instructions (ELISA Deluxe Kits, BioLegend, Amsterdam, Netherlands). The quantification limits of the assays were 7.8 pg/mL.

Statistics: Since data often were not normally distributed, groups were compared by Kruskal–Wallis test followed by two-tailed Dunn’s multiple-comparisons test to correct for repeated testing by means of GraphPad Prism Software (Version 6.0.; GraphPad, San Diego, CA, USA). *P*-values <0.05 were considered statistically significant.

## Results

**Figure 1:**
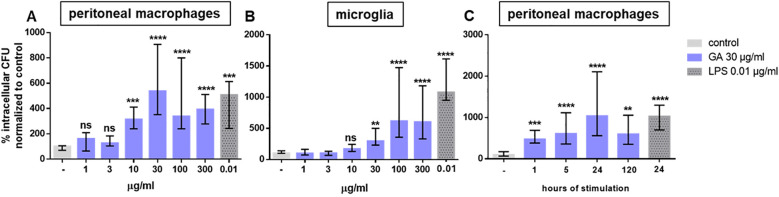
Dose- and time-dependent phagocytosis of *E. coli* by peritoneal macrophages (**(A)***n* = 18) and microglia (**(B)**, *n* = 9–14) isolated from young adult C57BL/6 mice. Macrophages **(A)** and microglial cells **(B)** were stimulated for 24 h by 1–300 µg/mL GA (blue) or 0.01 µg/mL LPS as a positive control (dark gray). **(C)** Peritoneal macrophages were stimulated by 30 µg/mL GA for 1–120 h (*n* = 9). Then, *E. coli* was co-incubated with eukaryotic cells for 60 min, and extracellular bacteria were killed by the addition of gentamicin for 60 min (antibiotic protection assay). The number of intracellular bacteria (colony forming units = CFU) was determined by quantitative plating on blood agar. Data are expressed as medians (25th/75th quartiles). Median phagocytosis of unstimulated cells in each individual experiment was defined as 100% (ns, not significantly different; ** *p* < 0.01, ***p < 0.001, ****p < 0.0001; Kruskal–Wallis test followed by Dunn’s multiple-comparisons test).

GA stimulated the phagocytosis of *E. coli* in both peritoneal macrophages and microglial cells in a dose-dependent manner (**Figures 1A, B**) without reducing the density of E. coli in the supernatants of the wells containing the macrophages or microglial cells, i.e., GA had no direct antibacterial activity against E. coli. In macrophages, 30 μg/mL of GA was most effective, whereas in microglia a concentration of 100 μg/mL was necessary to elicit a maximum effect (**Figures 1A, B**). In macrophages GA at 30 μg/mL was as potent as 0.01 μg/mL LPS, with both compounds increasing phagocytosis in median by approximately 500%. In microglial cells, GA at the most potent concentrations 100 and 300 μg/mL increased phagocytosis in median by approximately 600%, whereas LPS led to a median increase of the phagocytosis of *E. coli* of approx. 1,200%. In peritoneal macrophages, the increase of the phagocytosis by GA stimulation was time-dependent; the maximum effect was achieved after 24 h of stimulation (**Figure 1C**). For this reason, all further experiments were performed using a GA stimulus with a duration of 24 h.

**Figure 2:**
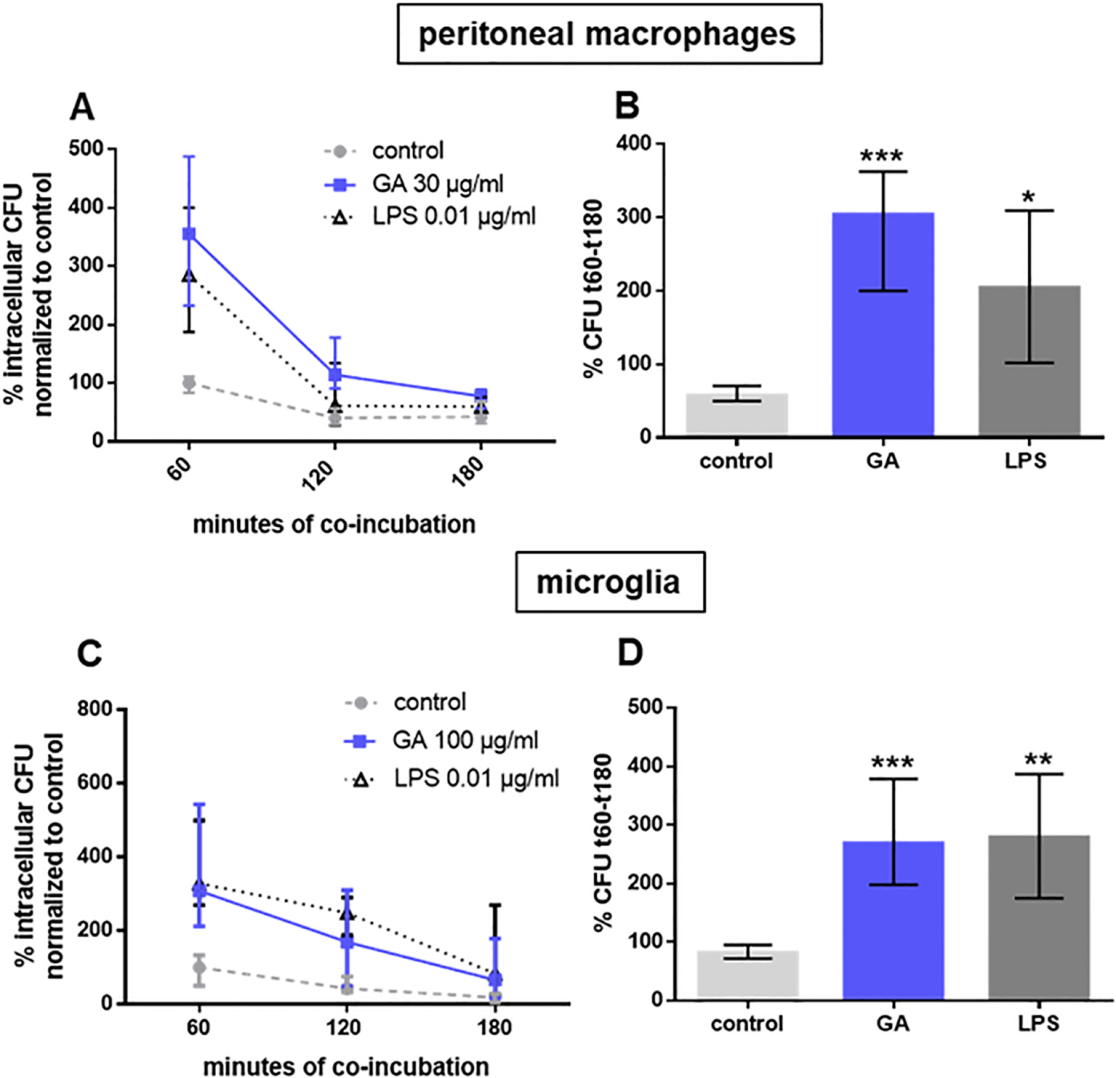
Intracellular killing of *E. coli* by peritoneal macrophages from young adult C57BL/6 (**(A, B)**; *n* = 9 to 10) and microglia prepared from newborn C57BL/6 mice (**(C, D)**; *n* = 8). Eukaryotic cells were co-incubated for 24 h with 30 (peritoneal macrophages) or 100 (microglia) µg/mL GA or 0.01 µg/mL LPS as a positive control. The number of intracellular bacteria was determined after 60 min of phagocytosis, followed by 60, 120, and 180 min of incubation with gentamicin to kill extracellular bacteria **(A, C)**. Median phagocytosis of unstimulated cells in each individual experiment was defined as 100%, and the percentage of intracellular bacteria at 180 min was subtracted from the percentage of intracellular bacteria at 60 min of gentamicin treatment **(B, D)**. Data are expressed as medians (25th/75th quartiles). *p < 0.05, **p < 0.01, ***p < 0.001; Kruskal–Wallis test followed by Dunn’s multiple-comparisons test.

**Figure 3:**
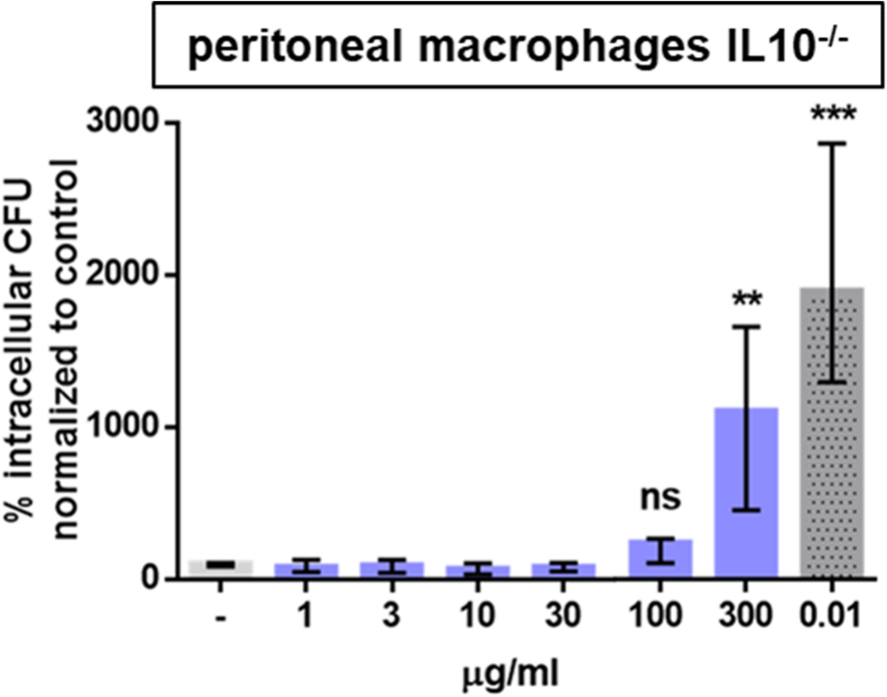
An increase in the phagocytic capacity of peritoneal macrophages isolated from young adult IL10^-/-^ mice was only detectable at concentrations of GA one order of magnitude higher than in wild-type macrophages. Eukaryotic cells were co-incubated for 24 h with 1–300 µg/mL GA or 0.01 µg/mL LPS as a positive control. An antibiotic protection assay was performed as described in **Figure 1**. Data are expressed as medians (25th/75th quartiles). Median phagocytosis of unstimulated cells in each individual experiment was defined as 100% (**p < 0.01, ***p < 0.001; *n* = 14, Kruskal–Wallis test followed by Dunn’s multiple-comparisons test).

**Figure 4:**
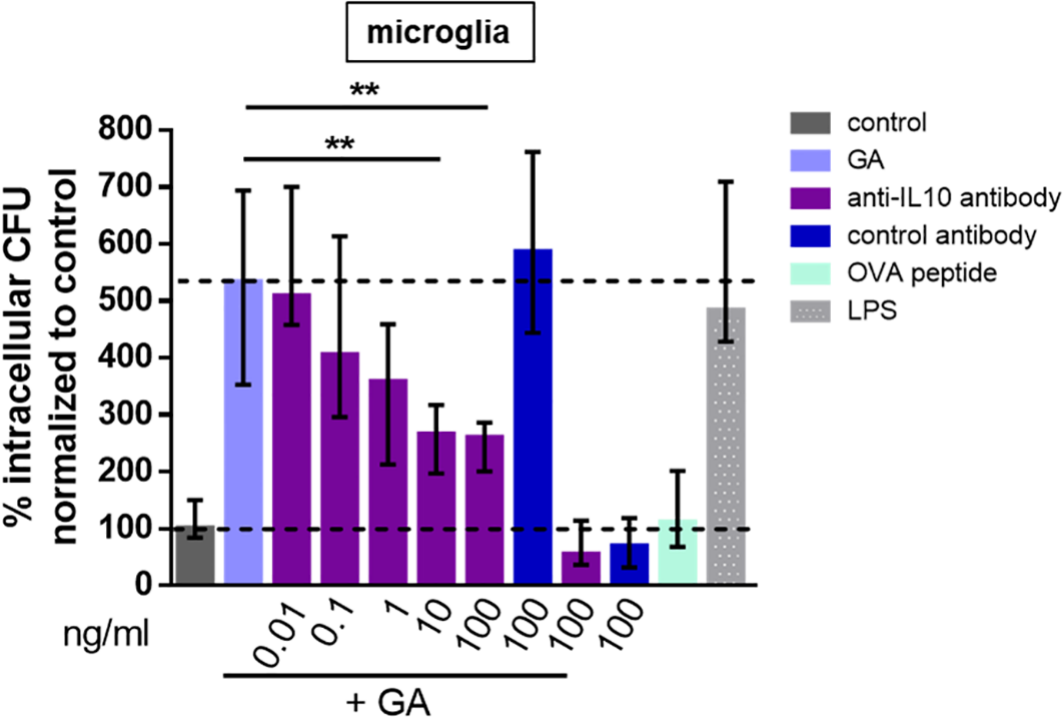
Blocking of IL10 decreased the GA-induced phagocytosis of *E. coli* by microglia from newborn mice in a dose-dependent manner. The microglia were stimulated with 100 µg/mL GA and incubated with 0.01–100 ng/mL anti-IL10 antibody or 100 ng/mL control antibody, with 100 µg/mL ovalbumin peptide as a negative and 0.01 µg/mL LPS as a positive control. An antibiotic protection assay was performed as described in **Figure 1**. Data are expressed as medians (25th/75th quartiles). Median phagocytosis of unstimulated cells in each individual experiment was defined as 100% (**p < 0.01; *n* = 4–8; Kruskal–Wallis plus Dunn’s multiple-comparisons test).

GA at the maximum effective concentration (30 μg/mL in peritoneal macrophages, 100 μg/mL in microglial cells) stimulated the ingestion and also the intracellular killing of phagocytosed *E. coli* (macrophages: **Figures 2A, B**; microglia: **Figures 2C, D**). In peritoneal macrophages prepared from IL10-deficient mice, a GA concentration of one order of magnitude higher than in wild-type macrophages was necessary to stimulate bacterial phagocytosis (**Figure 3**). In microglial cells, antibodies directed against IL10 inhibited the stimulatory effect of GA on *E. coli* phagocytosis in a concentration-dependent way. IL10-specific antibodies were not able to completely block the GA effect on phagocytosis but caused an inhibition in median of approximately 50% (**Figure 4**).

**Figure 5:**
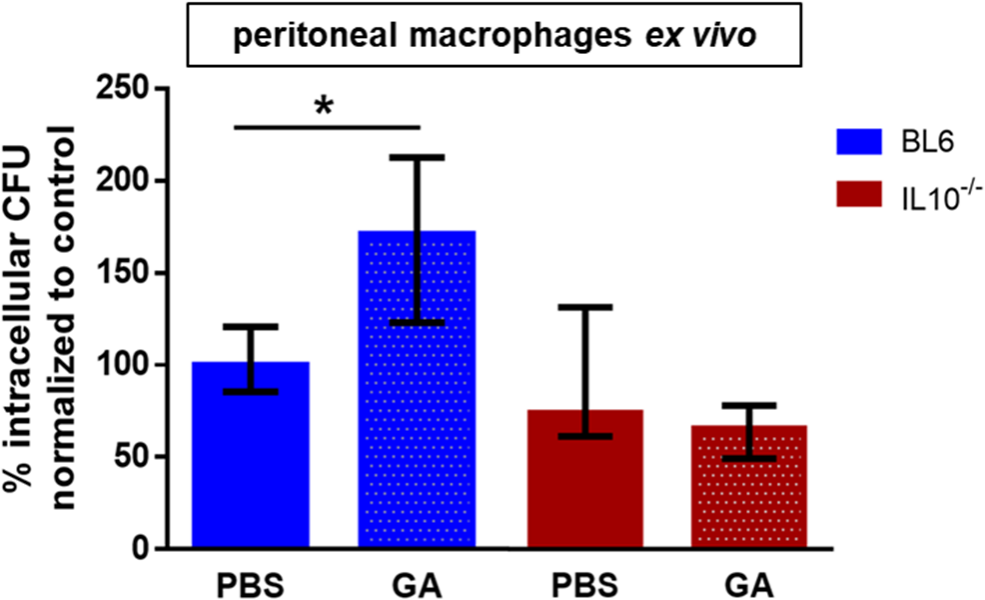
*Ex vivo* phagocytosis of *E. coli* by peritoneal macrophages from young adult mice was increased after *in vivo* treatment of mice with GA (blue). The increase of phagocytosis induced by GA depended on the presence of interleukin (IL) 10: in macrophages prepared from IL10-deficient mice, *in vivo* treatment of mice with GA did not stimulate phagocytosis (red). The 8-week-old male mice received subcutaneous injections of 150 µg GA per mouse once per day for five consecutive days. Then, peritoneal macrophages were isolated and cultivated for 24 h to perform an antibiotic protection assay as described in **Figure 1**. Data are expressed as medians (25th/75th quartiles). *p < 0.05, Kruskal–Wallis test followed by Dunn’s multiple-comparisons test; each group *n* = 5; BL6 = C57BL/6; IL10-/-, macrophages prepared from IL10-deficient mice.

Pre-stimulation of wild-type mice with GA prior to the preparation of macrophages (five doses at an interval of 24 h) caused an increase of phagocytosis of *E. coli* in median of approx. 70%. In IL10-deficient mice, pre-stimulation with GA did not increase the bacterial phagocytosis *ex vivo* (**Figure 5**).

Unlike LPS at a concentration of 0.01 µg/mL, incubation of peritoneal macrophages with GA at a concentration of 30 µg/mL for 24 h did not stimulate the release of TNFα and IL12/IL23 p40 (**Table 1**). Neither GA 30µg/mL nor LPS 0.01 µg/mL stimulation for 24 h released substantial amounts of IL12 p70 and γ-interferon from peritoneal macrophages (**Table 1**).

**Table 1:** Cytokine release (pg/mL) from peritoneal macrophages stimulated by glatiramer acetate (GA) at a concentration of 30 μg/mL for 24 h.

Stimulant	TNFα	IL12 p40	IL12 p70	γ-interferon
*n*	5	16	12–13	11
GA	bql (bql/bql)	bql (bql/33.7)	bql (bql/13.4)	bql (bql/53.7)
LPS 0.01 μg/mL	706.4 (639.4/1,164)	573.1 (266.3/667.3)	bql (bql/bql)	bql (bql/77.8)
Unstimulated control	bql (bql/bql)	bql (bql/38.5)	bql (bql/bql)	bql (bql/bql)
*p* (GA vs. control)	ns	ns	ns	ns
*p* (LPS vs. control)	0.0048	0.0007	ns	ns

Groups were compared by Kruskal–Wallis test followed by two-tailed Dunn’s multiple-comparisons test. ns, difference not statistically significant.

bql, measurement below the quantification limit of the assay (7.8 pg/mL); IL, interleukin; LPS, lipopolysaccharide from *Escherichia coli*; TNF, tumor necrosis factor.

**Figure 6:**
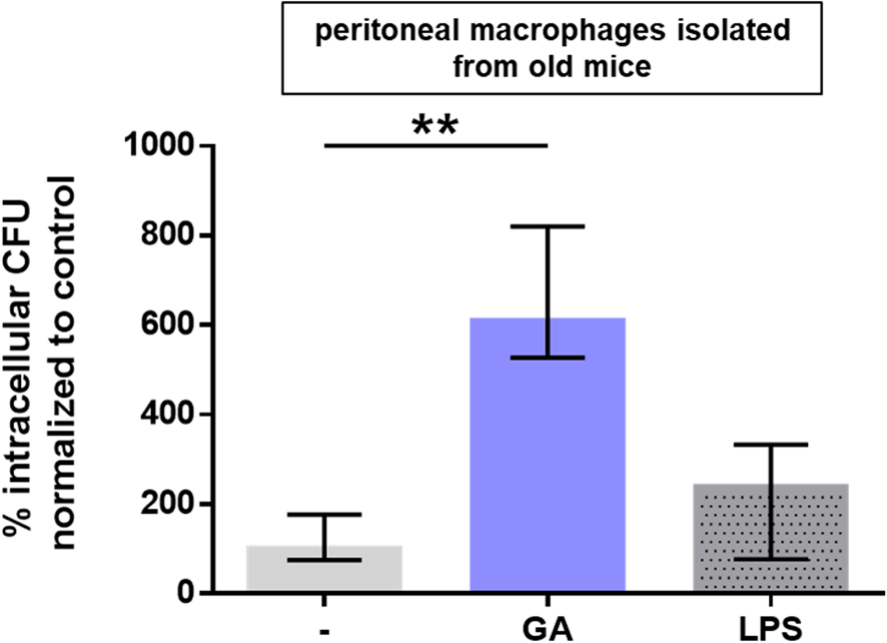
GA strongly stimulated the phagocytosis of *E. coli* in peritoneal macrophages prepared from 20-month-old mice (*n* = 6). The stimulatory effect of LPS on phagocytosis was less pronounced, and the differences failed to reach statistical significance. The median phagocytosis of unstimulated cells in each individual experiment was defined as 100% (ns, not significantly different; **p < 0.01; Kruskal–Wallis test followed by Dunn’s multiple-comparisons test).

GA did not only increase the phagocytosis of *E. coli* in young adult mice but also stimulated the phagocytosis of *E. coli* in peritoneal macrophages prepared from 20-month-old mice in median by 609% (*p* < 0.01, *n* = 6) (**Figure 6**). The stimulatory effect of LPS on bacterial phagocytosis in phagocytes from old mice was less pronounced (difference between LPS-stimulated and control macrophages not statistically significant).

## Discussion

Sepsis and meningitis are life-threatening diseases. Their incidence is highest in neonates, infants, elderly, and in immunocompromised persons of all ages. In these conditions, *E. coli* is one of the most frequent and virulent pathogens (21–23). For many of the pathogens causing sepsis and meningitis in immunocompromised patients, no vaccines are commercially available. The effectiveness of vaccination in immunocompromised patients is often reduced (24).

It has been reported previously that GA increased the phagocytosis of fluorescent beads *in vitro* (25, 26). The ingestion of beads was greater in monocytes from MS patients treated with GA than in those from healthy controls or untreated MS patients (25). In the present study, pre-stimulation of macrophages and microglial cells with GA increased their ability to ingest and kill pathogenic encapsulated *E. coli*. With respect to the patients’ safety and for the assessment of the immunomodulatory properties of GA, the finding that it does not only stimulate the phagocytosis of plastic beads but also the phagocytosis and killing of live bacteria is of great importance.

The age-related decline in immune functions (immunosenescence) comprises a decrease in cell-mediated and humoral immune responses. Old individuals are characterized by an increased basal level of pro-inflammatory cyto- and chemokines and the inability to mount an adequate immune response upon infection or vaccination (6). Therefore, many immunostimulants have a reduced or no activity on aged phagocytes (27). GA at low concentrations increased the phagocytosis of pathogenic *E. coli* not only in macrophages from young but also from old mice. This makes GA a promising candidate for the prophylaxis of infections in the elderly. Another compound which acts on phagocytes from young and old individuals—however, only at very high concentrations, which allows its use only as a local immunostimulant—is gum arabic (28). Unlike in a previous study (29), GA in our hands did not have a direct antibacterial effect: addition of GA did not result in a decreased number of bacteria in the supernatants of the cell culture wells.

Infectiologists are looking for single compounds increasing the infection resistance to various pathogens. One approach is the submaximal stimulation with epitopes or pathogen-associated molecular patterns (PAMPs). Not only the adaptive but also the innate immune system can be trained (30). In order to exert an optimum effect on the innate immune system, the stimuli must not be too strong: very strong or repeated stimuli (e.g., high or repeated doses of endotoxin or high concentrations of damage-associated molecular patterns (DAMPs)) induce immunosuppression (31, 32).

The innate immune memory is non-specific, has a shorter duration than the adaptive immune memory, and is mediated by epigenetic reprogramming in myeloid cells, natural killer cells, and γδ T cells (30, 33). Because bacterial DNA, unlike DNA from eukaryotic cells, possesses a high percentage of unmethylated cytosine–guanine (CpG) motifs (34), in recent years attention focused on this molecular pattern, a ligand of Toll-like receptor (TLR)-9. Stimulation of primary murine microglia with CpG oligonucleotides enhanced the phagocytosis of *E. coli* and *Streptococcus pneumoniae* and the intracellular killing of these pathogens (35, 36). Pre-treatment by CpG oligodeoxynucleotides in several models protected animals against a variety of bloodstream and other bacterial infections (35, 37–39). Not only bacterial but also the viral TLR agonist poly(I:C) can stimulate the phagocytosis and intracellular killing of bacteria (18). In a double injury model comprising cecal ligation and puncture (CLP) followed by intranasal infection with *Pseudomonas aeruginosa*, intranasal poly(I:C) treatment started 24 h after CLP led to a significant increase of survival (69% versus 33%) (40). Muramyl dipeptide (MDP) is the smallest peptidoglycan compound of both Gram-positive and Gram-negative bacteria with proinflammatory properties. It is a ligand of the nucleotide-binding oligomerization domain-like receptor 2 (NOD2) (41). It moderately activates microglial cells, increases the phagocytosis of bacteria, and acts in an additive or synergistic way with TLR agonists (19). MDP has been used successfully as an adjuvant for vaccination (42).

The prophylactic administration of stimulants of the innate immune system with the goal of increasing resistance to infections, as an adverse effect, can cause an unspecific inflammatory state. This may compromise an organism attempting to combat an infection: activated phagocytes do not only efficiently eliminate pathogens but can also acutely damage the host or lead to chronic autoimmune diseases (43). These phenomena are most devastating in the CNS (44–47). Therefore, activation of the TLR or NOD system aiming at increasing the resistance to infections bears the risk of inducing collateral damage to vessels, the nervous system, or other organs. In the search of compounds increasing the resistance to infection without inducing an inflammatory state, palmitoylethanolamide (PEA), an endogenous compound, represented a major advance. PEA possesses anti-inflammatory, neuroprotective, and analgesic properties (48). PEA pre-treatment increased the resistance of mice against exposure to *Shigella dysenteriae* toxin and streptolysin O, against intravenous infection with live group A streptococci and against intracerebral or intraperitoneal infection with *E. coli* (48, 49). Unlike TLR or NOD agonists and comparable to GA, PEA did not cause a release of TNFα, IL6, and CXCL1 by microglial cells (17). As GA, PEA therefore is no immunostimulant, but a true immunomodulator. Since PEA is marketed as a dietary supplement, however, probably no appropriate clinical study will be performed to prove or disprove its protective effect against infections in humans.

Unlike PEA, GA is available as a licensed drug in the majority of countries. It is well tolerated, even after treatment for many years. In monocyte-derived macrophages, HIV-1 virus production was reduced until day 10 in the presence of 50 μg/mL GA compared to untreated HIV-1 infected cells (16). In the absence of phagocytes, addition of GA to *E. coli*, *P. aeruginosa*, and *A. baumannii* cultures resulted in a retardation of growth (29). GA possessed antifungal properties with a half-maximal inhibitory concentration (IC50) of 0.470 mg/mL and a minimum inhibitory concentration (MIC) of 2.5 mg/mL (50), i.e., concentrations approximately three orders of magnitude higher than those of conventional antibiotics. Here we demonstrate that as with PEA (17, 49), GA increases the ability of macrophages and microglia to ingest and kill *E. coli* without stimulation of the release of the pro-inflammatory cytokines TNFα, IL12/IL23 p40, IL12 p70, and γ-interferon (present study). Since pro-inflammatory bacterial products injure axons and neuronal somata (45), a reduction of the bacterial load in the bloodstream during sepsis and in the CSF during meningitis by phagocytes prior or during simultaneous antibiotic therapy may help to reduce neuronal damage and sequelae in *E. coli* sepsis and meningitis.

In primary rat microglia, GA increased IL10 secretion, whereas it decreased the release of tumor necrosis factor-α (TNFα) and did not influence nitric oxide (NO) release. Treatment of monocytes with IL10 did not increase phagocytosis, but the addition of anti-IL10 receptor antibodies—as in the present study—decreased phagocytosis. The addition of anti-CD14, anti-CD16, anti-TIM-3, and anti-CD36 antibodies decreased phagocytosis by 30%–50%. The supernatants of stimulated monocytes did not increase phagocytosis in monocytes (25). GA acts on integrin macrophage-1 antigen (“complement receptor 3” consisting of CD11b and CD18) and on T-cell immunoglobulin mucin 3 (TIM-3), both present on macrophages (26). In human monocyte-derived macrophages, GA reduced TNFα production (51). Treatment of a human astrocytic cell line with GA inhibited the TNFα-induced mRNA and protein synthesis of RANTES in a dose- and time-dependent way (52). In the same human cell line, GA reduced the steady-state level of RANTES mRNA and blocked the IL1-dependent NF-κB activation and RANTES expression (53). Since GA resembles MBP, it appeared to bind as an antagonist to major histocompatibility (MHC) II complexes, thereby preventing myelin-derived antigens from presenting to T cells (54). GA further alters the differentiation of T cells and promotes T-helper 2 (Th2) compared to T-helper1 (Th1) cells (55). GA suppressed the STAT1/NF-κB pathways and promoted the release of the cytokines IL10 and TGFβ (51). GA also bound to the paired immunoglobulin (Ig)-like receptor-B in mice and to the human ortholog leukocyte immunoglobulin-like receptor-B. This interaction inhibited the STAT1 pathway and resulted in an increased IL10 and TGFβ secretion (51, 56).

Although IL10 is generally considered an anti-inflammatory cytokine, in murine models of intracerebral hemorrhage, microglial IL10 enhanced phagocytosis and accelerated the absorption of hematoma. As a consequence of delayed hematoma clearance, IL10-deficient mice had a stronger inflammatory response, edema, and iron deposition in the brain tissue and more severe neurologic deficits. The intranasal application of recombinant IL10 accelerated the absorption of the hematoma and improved the neurologic function. CD36 appeared to be a key phagocytosis effector regulated by IL10 (57). CD36 is a surface scavenger both recognizing DAMPs and PAMPs and also a long-chain free fatty acid transporter (58). In a murine model of Parkinson’s disease, microglia-specific expression of IL10 mitigated the excessive inflammatory response and transformed microglia into a molecularly distinct cell state with an enhanced expression of prophagocytic pathways. IL10 promoted microglial phagocytosis and reduced the load of α-synuclein aggregates in the substantia nigra (59). In bone marrow-derived macrophages *in vitro*, IL-10 promoted the phagocytosis of dysfunctional mitochondria (60). In cultured monocytes/macrophages, recombinant IL10 inhibited the replication of HIV, whereas it did not affect the lipopolysaccharide-induced production of IL1β, IL6, or TNFα (61). In an *in vitro* human whole-blood assay, IL10 strongly increased the monocytic CD14-dependent phagocytosis of *E. coli* and the CD14-dependent and CD14-independent phagocytosis of apoptotic cells (62). IL10 upregulated the expression of CD14 on monocytes. IL10 appears to activate several monocytic scavenger functions at least partly mediated by CD14 and to promote the differentiation of monocytes to macrophages (62). These findings illustrate how GA may act via IL10. In the defense against infections, GA does not have anti-inflammatory properties alone but, under appropriate conditions, stimulates the phagocytosis of detritus and—as in the present study—even pathogens. Further investigation into possible pathways and receptors involved is needed. It should be studied whether adding recombinant IL10 to IL10-deficient phagocytes increases their phagocytic ability after GA pretreatment, and the effect of blocking anti-IL10 receptor antibodies (in parallel to anti-IL10 antibodies used in the present study) could be studied in wild-type phagocytes. The anti-inflammatory and immunostimulatory properties of IL10 can be deconvoluted by IL10 variants with different binding strengths to the IL10 receptor subtypes (63). This may enable the design of agonists with specific properties such as increased infection resistance. In order to further explore the clinical impact of our study, the influence of GA on the phagocytosis of other Gram-negative and Gram-positive bacteria causing sepsis and meningitis in phagocytes prepared from young and old individuals should be assessed.

## Conclusion

As a consequence of the increased threat of patients by multi-resistant bacteria, agents with few side effects and a low toxicity, which are able to stimulate phagocytosis and the subsequent killing of pathogenic bacteria, are highly desirable. Due to its low toxicity even during long-term treatment and its ability to stimulate phagocytosis of bacteria not only in young but also in old mice, GA is an excellent candidate to increase the infection resistance of young and older persons, thereby preventing infections and reducing the amount of antibiotics prescribed. Before clinical trials in immunocompromised patients (e.g., old patients, patients treated in intensive care units or receiving immunosuppressants) can be started, our *in vitro* observation should be tested in adequate animal models.

## Data Availability

The raw data supporting the conclusions of this article will be made available by the authors, without undue reservation.
